# How can we realise the full potential of health systems for nutrition?

**DOI:** 10.1136/bmj.l6911

**Published:** 2020-01-26

**Authors:** Rebecca A Heidkamp, Emily Wilson, Purnima Menon, Helen Kuo, Shelley Walton, Giovanna Gatica-Domínguez, Inacio Crochemore da Silva, Tricia Aung, Nemat Hajeebhoy, Ellen Piwoz

**Affiliations:** 1 Department of International Health, Johns Hopkins Bloomberg School of Public Health, Baltimore, Maryland, USA; 2 Poverty, Health and Nutrition Division, International Food Policy Research Institute, Washington, DC, USA; 3 International Center for Equity in Health, Postgraduate Program in Epidemiology, Federal University of Pelotas, Pelotas, Brazil; 4 Nutrition Division, Global Development Program, Bill and Melinda Gates Foundation, Seattle, Washington, USA; Correspondence to: Rebecca Heidkamp rheidka1@jhu.edu

## Abstract

Poor nutrition contributes substantially to global disease, diminishing the wellbeing of women and children in low and middle income countries, and better nutrition must be part of the universal health coverage agenda, say **Rebecca Heidkamp and colleagues**

**Key messages**

- Most essential nutrition interventions are delivered through health systems
- Global movements to scale up effective nutrition interventions and achieve universal health coverage have not been connected to help each realise their full potential
- Scaling up nutrition interventions among those who are already reached by health services is an important first step for accelerating progress
- Other countries can learn from the experience of those that seem to be on track to achieving universal health coverage for specific health services and nutrition interventions
- We need to deal with the widespread gaps in data on the coverage of nutrition interventions if we want to monitor progress and achieve universal coverage

Over the past decade, global efforts to raise awareness of malnutrition have been accompanied by national movements to gain high level political commitment and accelerate actions, proved to work, to improve nutrition.^1^ Despite this momentum, by 2018 just under half of countries are on track to meet at least one of nine nutrition related targets set by the World Health Assembly for 2025 (table 1 ).^4^ Many countries are not achieving the goals because coverage of effective interventions remains low. Health systems are the primary vehicle for nutrition interventions in many low and middle income countries. Ideally, health systems reach women and children through frequent antenatal care, normal and emergency delivery services, early postnatal care, and preventive and curative care throughout early childhood. Nutrition interventions commonly include counselling about diets and infant and young child feeding during antenatal care, postnatal care, and for the first two years of life. Additionally, weight gain is monitored and nutritional supplements, particularly iron folic acid, provided during antenatal care. Common interventions for young children include high dose vitamin A supplements, growth monitoring and screening for, and treatment of, acute malnutrition, and treatment of childhood diarrhoea with zinc tablets in combination with oral rehydration solution.^5^

**Table 1:** Nutrition related goals in the global nutrition monitoring framework for maternal, infant, and young child nutrition^2^ and the non-communicable diseases global monitoring framework^3^

Target	Goal
Nutrition^2^	
1 Stunting	40% reduction in the number of children under-5 who are stunted
2 Anaemia	50% reduction of anaemia in women of reproductive age
3 Low birth weight	30% reduction in low birth weight
4 Childhood overweight	No increase in childhood overweight
5 Breastfeeding	Increase the rate of exclusive breastfeeding in the first 6 months up to at least 50%
6 Wasting	Reduce and maintain childhood wasting to less than 5%
Non-communicable diseases^3^	
4 Salt intake	30% relative reduction in mean population intake of salt/sodium
6 Adult raised blood pressure	25% relative reduction in prevalence of raised blood pressure or contain the prevalence of raised blood pressure, depending on national circumstances
7 Diabetes and obesity	No change in the prevalence of adult and adolescent diabetes, overweight and obesity

## Are health systems reaching target groups with nutrition interventions?

Gaps in data on the coverage of nutrition interventions are hampering efforts to evaluate progress, estimate benefits, and advocate further investments.^6^ Global household survey programmes, including the Demographic and Health Surveys and Multiple Indicator Cluster Surveys, have historically included a limited set of nutrition coverage indicators. These national sources enable aggregation and comparison of common indicators within and across countries. However, such data are available for less than half of the evidence based nutrition interventions recommended by the World Health Organization (table 2). Nevertheless, we can still use available data to identify opportunities to influence health systems for nutrition more effectively.

**Table 2:** Availability of household survey data (Demographic and Health Survey-7 or Multiple Indicator Cluster Survey-6 core questionnaires) on WHO recommended nutrition interventions delivered through health systems (adapted from Gillespie et al, *BMJ Global Health*, 2019)^6^

Stage	Data available	Data missing
Pre-pregnancy		• Folic acid • Iron supplements
Antenatal care	• Iron-folic acid supplements • Deworming	• Counselling about mother’s diet and use of supplements • Counselling and support for breastfeeding • Balanced energy protein supplements • Multiple micronutrient supplements • Calcium supplements • Vitamin A supplements
Delivery/early postnatal care	• Support for early initiation of breastfeeding at delivery • Counselling and support for exclusive breastfeeding (first two days only)	• Delayed cord clamping • Post partum iron supplementation
Childhood preventive care	• Vitamin A • Receipt of micronutrient powders	• Screening for acute malnutrition • Counselling and support for exclusive and continued breastfeeding after the first two days • Counselling and support for complementary feeding • Food supplements • Receipt of supplements other than micronutrient powders (iron; multiple micronutrient supplements; preventive zinc)
Childhood recuperative care	• Zinc for diarrhoea (recommended with oral rehydration solution) • Receipt of ready-to-use therapeutic food • Receipt of ready-to-use supplementary food	• Coverage of community based management of acute malnutrition (treatment of those with confirmed severe acute malnutrition/moderate acute malnutrition)

We studied publicly available data for five evidence based nutrition interventions^7^ and their associated service delivery for maternal, newborn, and child care for 50 countries that had at least one Demographic and Health Survey or Multiple Indicator Cluster Survey from 2013 to 2018. We pooled countries by World Bank country income group. We characterised the delivery “opportunity gap” as the absolute difference between coverage of the nutrition intervention and coverage of the health service through which it is commonly delivered—for example, iron folic acid supplementation is the nutrition intervention delivered through antenatal care. We considered early initiation of breastfeeding to be a proxy for intervention by birth attendants at delivery to support timely breastfeeding.^8^ We characterised zinc for diarrhoea as a nutrition intervention and oral rehydration solution as the health service because not all episodes of diarrhoea require contact with a health provider but all children who receive oral rehydration solution should also receive zinc.

We also examined coverage equity gaps in two ways. Firstly, we looked at absolute differences in coverage across rural and urban areas; secondly, we examined subnational variability in coverage by comparing the region with highest coverage with the region with lowest coverage. We examined trends by income group using countries that had at least one additional survey between 2008 and 2012. Indicator definitions, detailed methods, a discussion of limitations, and country data are available in the web supplement.

**Fig 1:**
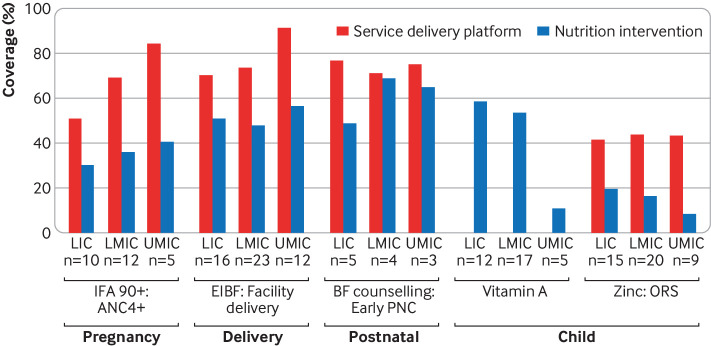
Pooled estimates of coverage of nutrition interventions and their respective health service delivery platforms by World Bank income group among countries with nationally representative Demographic and Health or Multiple Indicator Cluster Household surveys between 2013 and 2018. ANC=antenatal care; BF=breastfeeding; EIBF=early initiation of breastfeeding; IFA=iron folic acid; LIC=low income countries, LMIC=lower middle income countries; n=number of countries contributing to the pooled estimate; ORS=oral rehydration solution; PNC=postnatal care; UMIC=upper middle income countries

Several findings stand out. Firstly, closing the opportunity gap by increasing nutrition intervention coverage among those already reached by health services should be an immediate priority. The coverage of most nutrition interventions falls far below the reach of health services through which they are delivered, particularly for antenatal care and delivery care (fig 1). Closing this gap is essential to reach targets. A recent study from Malawi using national data estimated that if all women who reported antenatal care visits had received all recommended nutrition interventions, including iron folic acid, and counselling on appropriate nutrition during pregnancy and optimal breastfeeding, the prevalence of low birthweight would have had a relative 21% decrease, from 14% to 11%, and early initiation of breastfeeding would improve by almost 10 percentage points, from 76% to 86% (Joseph et al, unpublished data).

Secondly, children are being left behind. The reach of nutrition services targeted at children is falling far behind that for women. For example, zinc for diarrhoea treatment in children had the lowest coverage among the interventions we examined. Nearly 15 years after the release of the WHO recommendation for zinc to treat diarrhoea and multiple “calls to action” by leading public health researchers,^9^ coverage among children with diarrhoea is only 16% (fig 1).

**Fig 2:**
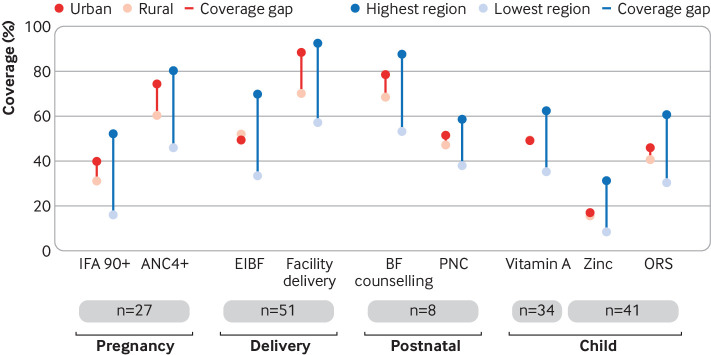
Pooled estimate of coverage of nutrition interventions and their respective health service delivery platforms by equity stratifier (rural/urban, high/low regional coverage) among countries with nationally representative Demographic and Health or Multiple Indicator Cluster household surveys between 2013 and 2018. ANC=antenatal care; BF=breastfeeding; EIBF=early initiation of breastfeeding; IFA=iron folic acid; n=number of countries contributing to the pooled estimate; ORS=oral rehydration solution; PNC=postnatal care

Thirdly, some subpopulations are being left behind. Rural coverage generally lags behind urban coverage but disparities are not large. The biggest coverage disparities within countries are between subnational regions (fig 2). This can be seen clearly in Cambodia, where the equity gap between rural and urban areas for iron folic acid is less than three percentage points but the equity gap between the provinces of Cambodia with highest and lowest iron folic acid coverage is 55 percentage points.

## Are we headed in the right direction?

**Fig 3:**
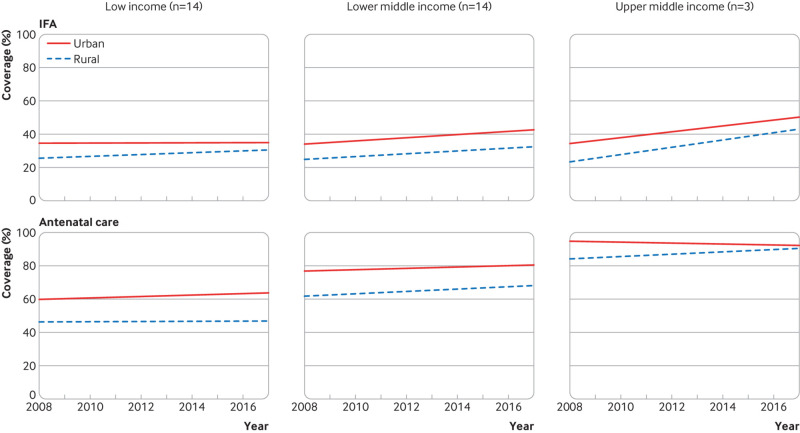
Comparison of 10 year trends (2008-18) between rural and urban coverage of iron folic acid supplementation and antenatal care by World Bank income group among countries with at least one nationally representative Demographic and Health or Multiple Indicator Cluster household survey in 2008-12 and 2013-18

**Fig 4:**
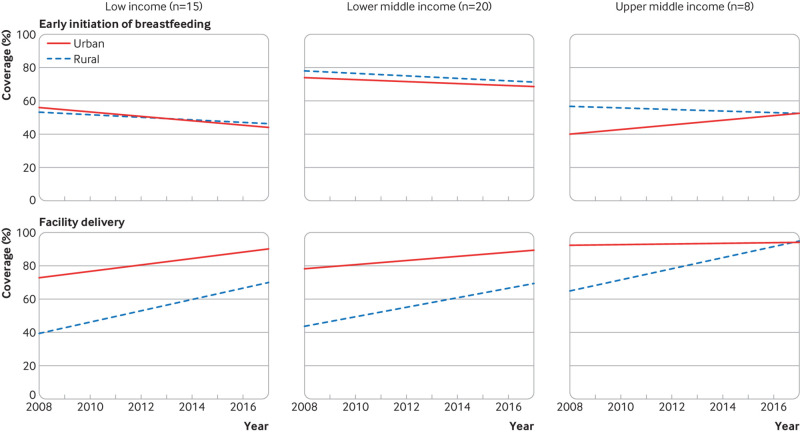
Comparison of 10 year trends (2008-18) between rural and urban coverage of early initiation of breastfeeding and facility delivery by World Bank income group among countries with at least one nationally representative Demographic and Health or Multiple Indicator Cluster household survey in 2008-12 and 2013-18

**Fig 5:**
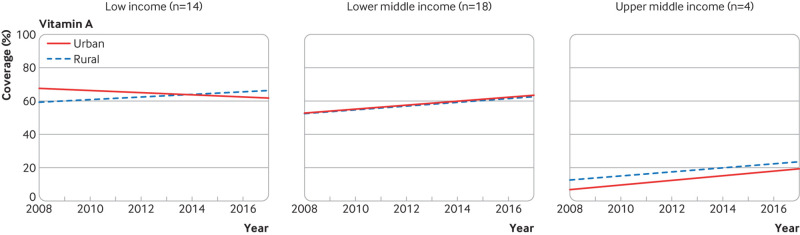
Comparison of 10 year trends (2008-18) between rural and urban coverage of vitamin A by World Bank income group among countries with at least one nationally representative Demographic and Health or Multiple Indicator Cluster household survey in 2008-12 and 2013-18

**Fig 6:**
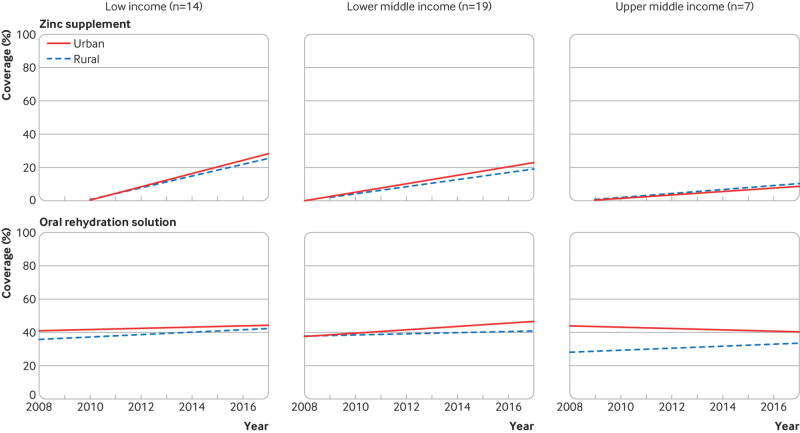
Comparison of 10 year trends (2008-18) between rural and urban coverage of zinc supplementation and oral rehydration solution among children with diarrhoea by World Bank income group among countries with at least one nationally representative Demographic and Health or Multiple Indicator Cluster household survey in 2008-12 and 2013-18

Coverage of nutrition interventions across country income groups is similar (fig 1). The exception is high dose vitamin A supplementation, which is not universally distributed in upper middle income countries, presumably owing to lower rates of deficiency. However, progress—defined by increasing coverage and decreasing inequality over time—varies by both intervention and income group. Figures 3-6 show the variability in progress across country income groups, comparing changes in rural and urban coverage of nutrition interventions and their associated health services over the past decade.

The opportunity gap between antenatal care and iron supplementation during pregnancy has reduced in lower middle income countries; however, the rural-urban equity gap remains. Coverage of both iron folic acid and antenatal care is stagnant in urban areas of low income countries, with slow improvements in iron folic acid coverage in rural areas. The opportunity gap is closing rapidly in upper middle income countries, but this seems to be because antenatal care coverage is stagnating, particularly among urban populations, even as iron folic acid coverage improves (fig 3).

Births in health facilities are increasing and the rural-urban equity gap has narrowed, with gains in rural areas. At the same time, we observe sharp declines in early initiation of breastfeeding, particularly among urban populations in low income countries (fig 4). The reasons for this are not clear. Increases in caesarean births may contribute to the decline in early initiation of breastfeeding in lower and upper middle income countries but caesarean births remain low overall in sub-Saharan Africa.^10^ A recent analysis of data from 2005 to 2017 shows that early initiation of breastfeeding increased only in countries where the coverage of institutional births improved by at least 20 percentage points.^11^

Questions on coverage of breastfeeding counselling during early postnatal care were only added to the Demographic and Health Surveys and Multiple Indicator Cluster Surveys in 2015, thus precluding analyses of trend. Among the few countries with available data for 2013-18, however, we saw that 75% of women received breastfeeding counselling in the first two days whereas only 60% reported an early postnatal visit with a health provider. Higher coverage of breastfeeding counselling may reflect the support provided before hospital discharge, a key tenet of the Baby-Friendly Hospital initiative.^8^ These figures also highlight the limits of our data. The standard survey questions do not provide us with the exact timing or location of early postnatal breastfeeding counselling.

The range of strategies for delivery of vitamin A—twice yearly campaigns, routine services, bundling with immunisation—makes it difficult to characterise the opportunity gap in the same way as for the other interventions. Although our analysis shows that high dose vitamin A supplementation coverage is similar between urban and rural areas (fig 2), 10 year trends show declines in urban populations of low income countries (fig 5). Once heralded as a successful public health nutrition programme, it is particularly alarming that coverage has slipped by the greatest amount in sub-Saharan African countries. These countries with high child mortality are precisely the populations that would benefit most. Some attribute this decline to use of campaigns, which are subject to delay or cancellation, or concerns about overlap with other vitamin A programmes, including fortified foods.^12^ ^13^

Zinc coverage has increased across countries, particularly low income countries, but remains unacceptably low (fig 6).

## Is there any good news?

Data show that some countries have successfully closed opportunity gaps, achieving high nutrition coverage and equity. For example, Senegal closed the opportunity gap for iron-folic acid during antenatal care. In 2017, iron folic acid coverage (63%) exceeded coverage of antenatal care delivery (56%). This runs counter to the typical pattern where iron-folic acid coverage is usually lower than that of antenatal care. Rwanda is noticeably different from other countries, with high early initiation of breastfeeding (81%) and high levels of facility delivery (91%) in 2014. The high coverage in this historically rural, but rapidly urbanising, country, also assures equity. 

In many other countries, however, large opportunity gaps are seen between facility based delivery and early initiation of breastfeeding. Countries with high rates of facility delivery but low early initiation of breastfeeding include Congo (92% facility delivery; 25% early initiation of breastfeeding); Egypt (87% facility delivery; 27% early initiation of breastfeeding), and El Salvador (98% facility delivery; 42% early initiation of breastfeeding). Countries that achieved high coverage of both postnatal care and breastfeeding counselling include the Philippines (postnatal care 86%; breastfeeding counselling 88%) and Jordan (postnatal care 87%; breastfeeding counselling 81%). Some countries have much higher rates of breastfeeding counselling than of postnatal care (Malawi 2015; Tanzania 2015; Angola 2015), suggesting possible use of other means of delivering counselling. Administrative data confirm a general drop in high dose vitamin A supplementation in most countries, but a few countries, including Zimbabwe, Kenya, and Sudan, do show improvements.^12^

## Leveraging the momentum around universal health coverage

In 2012, the UN General Assembly endorsed a resolution on universal health coverage, declaring that all people will have access to affordable, quality essential preventive and curative health services across their lifespans. Universal health coverage was enshrined in the 2015 sustainable development goals, and momentum for it has been building, including a high level event at the 2019 UN General Assembly.

Delivery of many nutrition interventions depends on a strong health system. However, despite parallel trajectories and shared concerns about slow progress in expanding coverage of interventions, discussions of nutrition and universal health coverage have been disconnected. Nutrition services for women and children are not included in the monitoring frameworks and indices of progress for universal health coverage.^14^ Until quite recently, there has been limited discussion among nutritionists of how universal health coverage can affect scaling up of proven nutrition interventions.^15^ ^16^

Unless countries leverage the momentum and invest in scaling up nutrition interventions as an integral component of universal health coverage, progress will remain off track. Key recommendations include:

*Provide health services for mothers and children—*Delivery needs to be accelerated to achieve gains in universal health coverage and nutrition. We found declines in coverage for key services (eg, antenatal care in upper middle income countries) and nutrition interventions, including high dose vitamin A supplementation and early initiation of breastfeeding. The reasons for these declines—and the actions needed to reverse them—may vary by context and require further investigation.
*Learn from exemplars—*Encouragingly, a range of countries offer examples of progress in scaling up health services, of integrating nutrition interventions within them, and of reaching marginal groups. Our empirical analyses do not provide insights into these improvements. In some cases, however, other reports can help us to understand how change occurs.^17^ For example, a study on successes in scaling up nutrition interventions in the Indian state of Odisha highlighted the roles of technical and administrative leadership, of financing models that offered stability, adequacy, and flexibility, of the use of data and routine reviews, and more.^18^ Disseminating such information enables policy makers to identify both common and uncommon contributors to success.
*Invest in data systems—*Our work shows the power of data. Data are a necessary, but not sufficient, ingredient for change in health systems. For example, to help to close opportunity gaps, countries should set targets for nutrition intervention coverage equal to those for their associated health services. However, without data, the effect of investments on improving intervention coverage cannot be accurately measured. Subnational data to identify and deal with geographical equity gaps are also lacking. Some signs of progress have been seen. In October 2019 the Demographic and Health Surveys programme released updated DHS-8 core questionnaires with considerably more questions on nutrition. Efforts are continuing, headed by Unicef, to develop core administrative data system modules for nutrition services. Without these data we cannot say whether universal health coverage is being attained.

Universal health coverage means quality care for all. Health services and nutrition interventions are targeting the same mothers and children. It is time to hold health systems accountable for their essential role in dealing with malnutrition.

## Web extra

Extra material supplied by authors

Extra information on methods

Data used for the analyses

## Notes

Contributors and sources: This article is based on data on coverage of interventions collated mostly from Demographic and Health and Multiple Indicator Cluster surveys from countries. RAH, PM, and EP conceived the outline of this paper and drafted the first versions. GG-D and ICdS provided collated data for multiple indicators. EW and HK collated additional data and conducted analysis of data. RAH, TA, and EW developed data visualisations. NH, SW, and TA reviewed, and revised drafts of the manuscript. RAH, PM, EP, and EW wrote the final version. RAH is the guarantor.

Provenance and peer review: Commissioned; externally peer reviewed.

This article is part of a series proposed by the Partnership for Maternal, Newborn and Child Health (PMNCH) hosted by the World Health Organization and commissioned by *The BMJ*, which peer reviewed, edited, and made the decisions to publish. Open access fees are funded by the Bill and Melinda Gates Foundaton and PMNCH.
