# Supplementary material for: How can we realise the full potential of health systems for nutrition?
Source: BMJ. 2020 Jan 26;368:l6911. doi: 10.1136/bmj.l6911 (PMC7461910; doi:10.1136/bmj.l6911)
Supplement: Supplementary file 1 — Extra information on methods [file heir51691.ww1.pdf]

Supplementary Materials 1: Additional information about methods

For all national coverage estimates we calculated survey-weighted indicators according to the definitions in Supplementary Materials Table 1 below. Estimates were stratified by geographic region and urban/rural residence using variables available in the Demographic and Health Surveys (DHS) & Multiple Indicator Cluster Surveys (MICS) datasets.

We generated pooled estimates for all countries and by World Bank Income Group by taking an unweighted mean of the country-specific estimates.

For current estimates (2013-2018) of intervention coverage (Figures 1 and 2) we included countries which had either a DHS or a MICS with estimates for both the nutrition intervention indicator and the associated health service indicator. If countries had more than one survey in 2013-2018, we used the most recent estimate.

For each World Bank country income group we described the 10-year trend in coverage of the interventions and delivery platforms across rural and urban areas between 2008-2018 (Figure 3-6) by fitting linear (OLS) models using survey year, rural-urban stratifier, and an interaction between year & rural-urban stratifier. We included the subset of countries which had at least one DHS or MICS in each of two time periods: 2008 to 2012, and 2013 to 2018 and included all available estimates for each country across that period.

In cases where World Bank country income group changed over the course of the analysis period – for example Albania which was classified as a "Lower-middle income" country in 2008, and an "Upper-middle income" country in 2017 – we used the most recent income group assignment.

Analyses were conducted in Stata, R, and Excel.

Limitations: Some limitations of the data we used for this analysis deserve mention. First, we were limited to inclusion of countries that have a DHS or MICS survey and as such results are not representative of all countries within an income group. Also The number of countries is particularly small and selective for UMIC and should be interpreted with care. surveys are carried out approximately every 3-5 years cut actual timing varies widely across countries, making it difficult to assess microvariations over time, which may be occurring. Second, DHS and MICS rely on maternal recall over variable periods of up to 3 years and may be subject to recall error. Sampling frames for most surveys included in this analysis were not designed to be sub-nationally representative. Finally, our coverage indicators do not reflect quality of services delivered, and although work is underway to develop quality-adjusted nutrition coverage measurements for nutrition.

Supplementary Table 1: Indicator Definitions *(green denotes nutrition intervention; white shading denotes health service platform)*

Variable name	***Indicator name***	***Numerator***	***Denominator***
anc4	Antenatal care (four or more visits)	Number of women ages 15–49 who were attended four or more times during the pregnancy that led to their last birth in the X years preceding the survey by any provider	Total number of women ages 15–49 with a live birth in the X years preceding the survey (X= 2 for MICS; 3 for DHS)
ironpreg	Iron supplementation during pregnancy (took 90+days)	Number of pregnant women who received the recommended number of iron/folic acid tablets (at least 90) during last pregnancy	Total number of interviewed women who had one or more live births in the X years preceding the survey (X= 2 for MICS; 5 for DHS)
ideliv	Facility delivery	Number of interviewed women who had one or more live births in a public or private health facility in the X years preceding the survey	Total number of interviewed women who had one or more live births in the X years preceding the survey (X= 2 for MICS; 5 for DHS)
bfearly	Early initiation of BF	Number of women with a live birth in the 2 years prior to the survey who put the newborn infant to the breast within one hour of birth	Total number of women with a live birth in the 2 years prior to the survey
pncbaby	Postnatal care for all babies	Number of last live births in the 2 years who received a health check while in facility or at home following delivery, or a postnatal care visit within 2 days after delivery	Total number of last live birth the 2 years prior to the survey (regardless of place of delivery)
bfco	BF counseling **OR** observation	Number of women age 15-49 with a live birth in the 2 years before the survey who were observed OR counseling about breastfeeding by a health provider in the first 2 days after delivery	Total number of women age 15-49 with a live birth in the 2 years before the survey
vita	Vitamin A supplementation for children	Number of children age 6-59 months who received vitamin A in the past 6 months	Total number of children ages 6-59 months
ors	ORS	Number of children ages 0–59 months with diarrhoea in the two weeks prior to the survey receiving oral rehydration salts	Total number of children ages 0–59 months with diarrhoea in the two weeks prior to the survey
zincd	Zinc treatment for diarrhoea	Number of children ages 0–59 months with diarrhoea in the two weeks prior to the survey receiving zinc	Total number of children ages 0–59 months with diarrhoea in the two weeks prior to the survey

Supplementary Table 2: Surveys contributing to Figure 1-2 current period estimates (2013-2018) by year, survey type and indicator

Income Group	Country	Year	Survey Type	Indicator								
				anc4	bfco	bfearly	ideliv	ironpreg	ors	pncbaby	vita	zincd
Low income (LIC)	Afghanistan	2015	DHS	X		X	X	X	X		X	X
	Bangladesh	2014	DHS			X	X		X		X	X
	Benin	2014	MICS			X	X		X		X	X
	Cambodia	2014	DHS	X		X	X	X	X		X	X
	Chad	2014	DHS	X		X	X	X	X		X	X
	Malawi	2015	DHS	X	X	X	X	X	X	X	X	X
	Mali	2015	MICS			X	X		X			X
	Nepal	2014	MICS			X	X					
	Rwanda	2014	DHS	X		X	X	X			X	
	Sierra Leone	2017	MICS			X	X		X			X
	Tajikistan	2017	DHS	X	X	X	X	X	X	X	X	X
	Tanzania	2015	DHS	X	X	X	X	X	X		X	X
	Zimbabwe	2015	DHS	X	X	X	X	X	X	X	X	X
Lower Middle Income (LMIC)	Armenia	2015	DHS	X	X	X	X	X	X	X		X
	Cameroon	2014	MICS			X	X		X		X	X
	Congo Republic	2014	MICS			X	X		X			X
	Egypt	2014	DHS	X		X	X	X	X		X	X
	El Salvador	2014	MICS			X	X		X		X	X
	Eswatini	2014	MICS			X	X		X		X	X
	Ghana	2014	DHS	X		X	X	X	X		X	X
	Guatemala	2014	DHS	X		X	X	X	X		X	X
	Guyana	2014	MICS			X	X					
	Lesotho	2014	DHS	X		X	X	X	X		X	X
	Pakistan	2017	DHS	X	X	X	X	X	X	X	X	X
	Philippines	2017	DHS	X	X	X	X	X	X	X	X	X
	Sao Tome and Principe	2014	MICS			X	X				X	
	Senegal	2017	DHS	X	X	X	X	X	X	X	X	X
	State of Palestine	2014	MICS			X	X					
	Sudan	2014	MICS			X	X		X		X	X
	Kenya	2014	DHS	X		X	X	X	X		X	X
	Kyrgyzstan	2014	MICS			X	X		X			X
	Mauritania	2015	MICS			X	X		X			X
Upper-middle income (UMIC)	Albania	2017	DHS	X	X	X	X	X	X	X		X
	Belize	2015	MICS			X	X		X			X
	Iraq	2018	MICS		X	X	X		X			X
	Colombia	2015	DHS									
	Cuba	2014	MICS			X			X			X
	Jordan	2017	DHS	X	X	X	X	X	X	X	X	X
	Kazakhstan	2015	MICS			X	X					
	Peru	2015	NSS	X		X	X	X	X		X	X
	Serbia	2014	MICS			X	X					
	Thailand	2015	MICS			X	X					

Supplementary Table 3: Surveys contributing to Figure 3-6 trend estimates (2008-2018) by year, survey type and indicator

Income Group	Country	Period 1 (2008-2012)									Period 2 (2013-2018)								
		Survey Year	Survey Type	anc4	ironpreg	ideliv	bfearly	vita	ors	zincd	Survey Year	Survey Type	anc4	ironpreg	ideliv	bfearly	vita	ors	zincd
Low income (LIC)	Afghanistan	2010	MICS			X	X	X	X	X	2015	DHS	X	X	X	X	X	X	X
	Benin	2011	DHS	X	X	X	X	X	X	X	2014	MICS			X	X	X	X	X
	Cambodia	2010	DHS	X	X	X	X	X	X	X	2014	DHS	X	X	X	X	X	X	X
	Chad	2010	MICS			X	X	X	X	X	2014	DHS	X	X	X	X	X	X	X
	Malawi	2010	DHS	X	X	X	X	X	X	X	2015	DHS	X	X	X	X	X	X	X
	Mali	2009	MICS			X	X	X			2015	MICS			X	X		X	X
		2012	DHS	X	X	X	X	X	X	X									
	Mozambique	2008	MICS			X	X				2015	AIS						X	X
		2011	DHS	X	X	X	X	X											
	Nepal	2011	DHS	X	X	X	X	X	X	X	2014	MICS			X	X			
	Rwanda	2010	DHS	X	X	X	X	X			2014	DHS	X	X	X	X	X		
	Senegal	2010	DHS	X	X	X	X	X	X	X	2015	DHS	X	X	X	X	X	X	X
		2012	DHS	X	X	X	X	X	X	X	2017	DHS	X	X	X	X	X	X	X
	Sierra Leone	2008	DHS	X	X	X	X	X	X	X	2017	MICS			X	X		X	X
		2010	MICS			X	X	X	X	X									
	Tajikistan	2012	DHS	X	X	X	X	X	X	X	2017	DHS	X	X	X	X	X	X	X
	Tanzania	2010	DHS	X	X	X	X	X	X	X	2015	DHS	X	X	X	X	X	X	X
	Zimbabwe	2009	MICS			X	X	X			2014	MICS			X	X	X	X	X
		2010	DHS	X	X	X	X	X	X	X	2015	DHS	X	X	X	X	X	X	X
Lower-middle income (LMIC)	Armenia	2010	DHS	X	X	X	X		X	X	2015	DHS	X	X	X	X		X	X
	Bangladesh	2011	DHS			X	X	X	X	X	2014	DHS			X	X	X	X	X
		2012	MICS			X	X		X	X									
	Cameroon	2011	DHS	X	X	X	X	X	X	X	2014	MICS			X	X	X	X	X
	Congo Republic	2011	DHS	X	X	X	X	X			2014	MICS			X	X		X	X
	Egypt	2008	DHS	X	X	X	X	X	X	X	2014	DHS	X	X	X	X	X	X	X
	El Salvador	2008	RHS			X	X	X			2014	MICS			X	X	X	X	X
	Eswatini	2010	MICS			X	X	X			2014	MICS			X	X	X	X	X
	Ghana	2008	DHS	X	X	X	X	X	X	X	2014	DHS	X	X	X	X	X	X	X
		2011	MICS			X	X	X	X	X									
	Guatemala	2008	RHS			X	X	X			2014	DHS	X	X	X	X	X	X	X
	Guyana	2009	DHS	X	X	X	X		X	X	2014	MICS			X	X			
	Kenya	2008	DHS	X	X	X	X	X	X	X	2014	DHS	X	X	X	X	X	X	X
	Kyrgyzstan	2012	DHS	X	X	X	X	X	X	X	2014	MICS			X	X		X	X

Income Group	Country	Period 1 (2008-2012) Survey Year	Survey Type	anc4	ironpreg	ideliv	bfearly	vita	ors	zincd	Period 2 (2013-2018) Survey Year	Survey Type	anc4	ironpreg	ideliv	bfearly	vita	ors	zincd
	Lesotho	2009	DHS	X	X	X	X	X			2014	DHS	X	X	X	X	X	X	X
	Mauritania	2011	MICS			X	X	X	X	X	2015	MICS			X	X		X	X
	Pakistan	2012	DHS	X	X	X	X	X	X	X	2017	DHS	X	X	X	X	X	X	X
	Philippines	2008	DHS	X	X	X	X	X	X	X	2017	DHS	X	X	X	X	X	X	X
	Sao Tome & Principe	2008	DHS	X	X	X	X	X	X	X	2014	MICS			X	X	X		
	Senegal	2010	DHS	X	X	X	X	X	X	X	2014	DHS	X	X	X	X	X	X	X
		2012	DHS	X	X	X	X	X	X	X									
	State of Palestine	2010	MICS			X	X	X			2014	MICS			X	X			
	Sudan	2010	MICS			X	X		X	X	2014	MICS			X	X	X	X	X
Upper-middle income (UMIC)	Albania	2008	DHS	X	X	X	X		X	X	2017	DHS	X	X	X	X		X	X
	Belize	2011	MICS			X	X	X			2015	MICS			X	X		X	X
	Cuba	2010	MICS						X	X	2014	MICS						X	X
	Iraq	2011	MICS			X	X	X	X	X	2018	MICS			X	X		X	X
	Jordan	2012	DHS	X	X	X	X	X			2017	DHS	X	X	X	X	X	X	X
	Kazakhstan	2010	MICS			X	X				2015	MICS			X	X			
	Peru	2008	DHS	X	X	X	X	X			2014	DHS	X	X	X	X	X	X	X
		2009	DHS	X	X	X	X	X	X	X	2015	DHS[1]	X	X	X	X	X	X	X
		2010	DHS	X	X	X	X	X	X	X									
		2011	DHS	X	X	X	X	X	X	X									
		2012	DHS	X	X	X	X	X	X	X									
	Serbia	2010	MICS			X	X				2014	MICS			X	X			
	Thailand	2012	MICS			X	X		X	X	2015	MICS			X	X			

[1] 2015 Peru DHS was a non standard survey
